# Metagenomics: Retrospect and Prospects in High Throughput Age

**DOI:** 10.1155/2015/121735

**Published:** 2015-11-17

**Authors:** Satish Kumar, Kishore Kumar Krishnani, Bharat Bhushan, Manoj Pandit Brahmane

**Affiliations:** ^1^ICAR-National Institute of Abiotic Stress Management, Baramati, Pune, Maharashtra 413115, India; ^2^ICAR-Central Institute of Post-Harvest Engineering and Technology, Abohar Station, Punjab 152116, India

## Abstract

In recent years, metagenomics has emerged as a powerful tool for mining of hidden microbial treasure in a culture independent manner. In the last two decades, metagenomics has been applied extensively to exploit concealed potential of microbial communities from almost all sorts of habitats. A brief historic progress made over the period is discussed in terms of origin of metagenomics to its current state and also the discovery of novel biological functions of commercial importance from metagenomes of diverse habitats. The present review also highlights the paradigm shift of metagenomics from basic study of community composition to insight into the microbial community dynamics for harnessing the full potential of uncultured microbes with more emphasis on the implication of breakthrough developments, namely, Next Generation Sequencing, advanced bioinformatics tools, and systems biology.

## 1. Introduction

Despite the exhaustive knowledge of intricate molecular mechanisms of most of the cellular processes and the availability of complex culture media, scientists are still able to culture less than 1% of all microorganisms present in diverse natural habitats. This leaves scientists unable to study more than 99% of the biological diversity in the environment with conventional techniques. Metagenomics is the function-based or sequence-based culture independent analysis of metagenomes trapped from a wide range of habitats. A typical metagenomic study combines the potential of genomics, bioinformatics, and systems biology in exploring the collective microbial genomes isolated directly from environmental samples. Course changing developments in recent times, like inexpensive Next Generation Sequencing (NGS) technologies, advanced bioinformatics tools, and high throughput screening (HTS) methods for metagenomic libraries, have left greatest impact on the science of metagenomics. These breakthrough developments have set a wave of excitement among large number of research groups all across the globe, triggering strong quest about the concealed potential of the existing microbial world beyond Petri dish. The cost of the large scale sequencing has reduced dramatically in the last few years. Using NGS, now it has become routine to generate hundreds of megabases of sequence data for expense of well under $20,000 bringing metagenomics in reach of many laboratories across the globe [1]. These advances in sequencing technologies have fuelled the research on metagenomics and have laid the way for the scientific community to undertake mammoth projects generating huge amount of sequence data. Dinsdale et al. [2] in their study on metagenomic comparison of 45 distinct microbiomes and 42 viromes generated 15 million sequences employing Next Generation Sequencing (NGS) and revealed strong discriminatory metabolic profiles across all the investigated microbiomes. Although the large scale sequencing studies in the pilot project on Sargasso Sea [3] and its extension, the Sorcerer II Global Ocean Sampling expedition [4], were carried out using Sanger sequencing based ABI 3750XL sequencer, Sanger sequencing is no longer the main source of metagenomic sequence data. The impact of NGS technologies on metagenomics has been so profound that a typical metagenomic project in the recent times generates large amounts of sequence data and due to this dominance of sequence-based projects, Kunin et al. [1] have redefined the metagenomics as “application of shotgun sequencing to DNA obtained directly from environmental sample producing at least 50 Mbp randomly sampled sequence data.” Metagenomic tools have allowed us the unprecedented access to the natural microbial communities and their potential activities. Metagenomics is now an established and prospered research arena and has completely suppressed the once prevailed erroneous notion that microorganisms did not exist unless they could be cultured. Initially, the research endeavours of most of the groups were primarily focused on answering the questions investigating “who are there” and have now shifted to finding key aspects of “what they are doing and how exactly they do it.” The present review summarizes the historic landmarks critical in the progression of the science of metagenomics and also highlights the progress made during the last two decades for trickling novel functions in metagenomes. This review also encompasses the impact of course changing developments in DNA sequencing and bioinformatics in the progression of science of metagenomics.

## 2. Metagenomics: Inception, Landmarks, and Progression

Though the term metagenome came off late in 1998 [5], the reports about unculturability of microbes go hundred years back to 1898, when Heinrich Winterberg first reported about microbial unculturability, the so-called great plate count anomaly. Owing to the lack of culture methods for a major segment of the microbes, their genetic potential remained unutilised for a longer time. Before 1985, most of what was known to us about the existence of microbial world was derived from cultured microbes. The studies of Staley and Konopka [6] in 1985 regarding the existing data of that time on “great plate count anomaly” highlighted first time the level of ignorance about microbial world and affirmed the fact that larger spectrum of microbes was left unaccessed. This affirmation of Staley and Konopka did not prove convincing to microbiologists of that time. Later, in 1990, studies of DNA-DNA reassociation kinetics of soil DNA by Torsvik et al. [7] provided the compelling evidence that culturing did not capture the complete spectrum of microorganism because the majority of microbial cells that could be seen in a microscope with various staining procedures could not be induced to produce colonies on Petri plates or cultures in test tubes. During this decade of 1980s, evidence started accumulating which drew attention of the scientific community towards uncultured microbial world, and the belief that microbial world had been conquered was laid to rest.

The pioneering work of Woese [8] in 1985 explicated that the 16S rRNA gene provides evolutionary chronometer and this proposal of Woese changed the whole progression of microbiology at that time. Development of PCR technology and primer designed to amplify the complete 16S rRNA gene left a catalytic effect and 16S rRNA gene became a phylogenetic marker of choice. Owing to its universal presence in all bacteria, its multigene nature, and its large enough size (1500 bp) for informatics purpose, the 16S rRNA gene marker has been employed most extensively for characterization of naturally occurring microbiota.

The idea that 16S rRNA gene from the environmental samples can directly be cloned was first put forward by Pace et al. in 1985 [9]. Later, in 1991, Schmidt et al. [10] reported successful cloning of 16S rRNA gene sequences from marine picoplankton communities using bacteriophage lambda vector. Though the cloning of 16S rRNA gene by Schmidt et al. was a breakthrough, the hidden metabolic potential of the community members could only be achieved by functional screening of cloned genes of metagenomic origin. Later, in 1995, Healy et al. [11] recovered the cellulose and xylosidase encoding genes by functional screening of metagenomic libraries from environmental DNA isolated from the mixed liquor of thermophilic, anaerobic digesters.

In the last two decades, all sorts of natural environments, for example, soils [12–17], marine picoplankton [18–20], hot springs [21–25], surface water from rivers [26], glacier ice [27], Antarctic desert soil [28], and gut of ruminants [29], have been targeted for metagenomic analysis. Initially, most of the studies carried out on metagenomic diversity analysis targeted at various sample types were based on traditional approaches, such as denaturing gradient gel electrophoresis (DGGE) [30], terminal restriction fragment length polymorphism (T-RFLP) analysis [31], or Sanger sequencing of 16S rRNA gene clone libraries [32]. Sanger sequencing of 16S rRNA gene was dominant approach from 1990 onwards and has been used extensively to access microbial community from almost every harsher environment. Widespread sequencing of ribosomal RNA genes has resulted in the generation of large reference databases, such as the ribosomal database project (RDP) II [33], Greengenes [34], and SILVA [35]. These comprehensive databases allow classification and comparison of environmental 16S rRNA gene sequences. Traditional surveys of environmental prokaryotic communities are based on amplification and cloning of 16S rRNA genes followed by sequence analysis. In the case of some bacterial communities which are amorphous in terms of phylogenetic relationship, 16S rRNA gene based studies have found that unsuitable and functional genes have been used for detection of such functional groups of microbes [36]. As compared to 16S rRNA genes, functional genes are shown to provide a greater resolution for the study of genetic diversity in natural populations of these bacterial communities. Whole community DNA based studies have been used to reveal microbial diversity of particular functional groups of microbes in environmental samples on the basis of functional gene markers. Many functional gene markers, namely, gene* soxB* (unique gene to sulphur oxidizing bacteria) [37] and ammonia monooxygenase,* amoA* (unique to ammonia oxidizing microbes) [38], have been applied to ascertain the diversity of these functional groups of microbes in environmental samples.

## 3. Prospecting Metagenomes: Towards Unlocking the Concealed Microbial Potential

Unculturable microbes cannot be isolated; hence their tremendous genetic potential can only be exploited by functional metagenomic approaches. Absence of an appropriate biocatalyst has been an impeding factor for many biotransformation processes. With advancement in basic molecular biology techniques, it is now possible to put metagenomics gene sequences from uncultured microbes into expression vectors which on subsequent expression produce novel peptides inside the host cells. Presence of novel proteins can be confirmed by screening the metagenomics clones displaying desired biological activity (function-based screening). Screening of metagenomic clones often involves a simple colour reaction mediated by the enzyme/biomolecule sought (product of cloned gene), which acts on a substrate linked to chromophores leading to the development of a certain colour pattern which is detected either visually or spectrophotometrically.

In the last two decades, many novel antibiotics, drugs, and enzymes/isozymes have been recovered from metagenomic libraries constructed from various environmental samples (Table 1). Constructing metagenomic libraries from environmental samples and subsequent cloning into the expression vectors followed by activity-based screening has endless possibilities of unlocking concealed potential in uncultured microbial world. The activity-based screening of metagenomic libraries initially suffered from low sensitivity and low throughput. Development of high throughput functional screen methods, namely, SIGEX (substrate induced gene expression) [39], METREX (metabolite regulated expression) [40], and PIGEX (product induced gene expression) [41], has accelerated isolation of novel biocatalysts from the environmental samples in last eight years. These high throughput screening methods employ the resolving power of FACS (fluorescence-activated cell sorting) or fluorescence microscopy. The fluorescence-activated cell sorting (FACS) is having wide application for high throughput screening of metagenomic clones, as it can be used to identify the biological activity within a single cell [42].

Limited availability of enzyme activity assay and narrow choice of host for transformation (most often* E. coli*) have been a main constraint in functional metagenomics research. In recent years, new transformation systems have been reported which use different microbes with alternative gene expression system and wide range of protein secretion mechanisms. Development of new host systems using microbes, namely,* Streptomyces* spp. [43],* Thermus thermophilus* [44],* Sulfolobus solfataricus* [45], and Proteobacteria [46], has widened the choice of host and compatible enzyme assay systems.* E. coli*, owing to its ease of transformation and being the best genetically characterised bacterium, has been the choice host for heterologous gene expression in metagenomic studies. With synchronised advances in the HTS (high throughput screening) methods and the choice of transformation systems with wide available range of hosts for heterologous gene expression, the field of functional metagenomics got tremendous momentum. It is now possible to screen up to 50,000 clones per second or over one billion clones per day using system developed by Diversa Corp. (now the part of BASF) which integrates laser with various wavelength capabilities, enabling mass screening of metagenomic clones [47].

These advances in functional metagenomics have paved industry with an unprecedented chance to bring biomolecules of metagenomic origin into a commercial success. Diversa Corp. remained the most prominent biotech company up to 2006 for commercialisation of technologies that evolved out of metagenomic research which was later merged with Celunol Corp. to create Verenium which was further merged with BASF. BASF and other major players like DSM, Syngenta, Genencor International, and BRAIN AG collaborated with different research groups and have commercialised many biological molecules of commercial interest (for details readers are directed to read review by Cowan et al. [48]). Expressing cloned genes of metagenomic origin in heterologous host enables researchers to access the tremendous genetic potential in a microbial community without knowing anything about the original gene sequence, the structure and composition of the desired protein, or the origin of microbe. Functional screening of metagenomic libraries constructed from environmental samples has been found to express interesting moonlighting protein (proteins having two different functions within a single polypeptide chain). Jiang et al. [49] in 2011 reported a novel *β*-glucosidase gene (bgl1D) with lipolytic activity (thus renamed as Lip1C) which was identified through function-based screening of a metagenomic library constructed from soil. Lipase and esterase remain the most targeted enzyme activities using functional screening of metagenomic libraries of diverse origin [50–55].

## 4. High Throughput Sequencing and Bioinformatics Tools: Adding New Dimensions to Metagenomics

The arrival of NGS (Next Generation Sequencing) technologies has left most profound impact on the metagenomics and has expanded the scale and scope of metagenomic studies in a way never imagined before. The first NGS technology, which could be materialized due to incredible amalgam of nanotechnology, organic chemistry, optical engineering, enzyme engineering, and robotics, became a viable commercial offering in 2005. The NGS platforms have been used for standard sequencing applications, such as genome sequencing and resequencing, and also for novel applications previously unexplored by Sanger sequencing. Before arrival of NGS platforms, Venter et al. [3] in 2004 generated high magnitude metagenomics sequence data to the tone of 1.66 million reads, comprised of 1.045 billion base pairs with an average read length of 818 bp from metagenomic samples collected from Sargasso Sea. In a further extension of the same endeavour during Sorcerer II Global Ocean Sampling expedition, Rusch et al. [4] generated 7.7 billion sequencing reads, comprising 6.3 billion base pairs using Sanger sequencing. This large amount of sequence data using Sanger sequencing was a great endeavour but the magnitude of data which are produced in a single run of NGS machine is severalfold higher. The large scale sequencing projects and consortia have already produced NGS derived huge sequence data sets, namely, The ENCODE project (over 15 trillion bases of raw data) [56], 1000 Genomes (over 20,000 Gb bases of raw data with about 5x coverage) [57], Human Microbiome Project (over 5 terabytes of genomic data) [58], and Earth Microbiome Project (envisage to produce over two petabytes of sequence data) [59]. The NGS platforms have paved the way to directly sequence the metagenomic DNA circumventing the need for tedious steps of cloning and library preparation. NGS platforms allow massive parallel sequencing where hundreds of thousands to hundreds of millions of sequencing reactions are performed and detected simultaneously, resulting in very high throughput. As multiple NGS platforms coexist in the market place with the unique chemistry of each, the decision about the suitability of a particular type of NGS platform for a metagenomic project is most critical in deciding the outcome of metagenomic studies. Hence, the selection of a particular NGS platform has to be made on the basis of varying features of NGS platforms like read length, degree of automation, throughput per run, data quality, ease in data analysis, and cost per run as compared in Table 2 (for details readers are directed to read the review by Liu et al., 2012 [60]).

454/Roche Life Sciences (pyrosequencing technology) and the Illumina/Solexa system are two most extensively applied sequencing platforms for metagenomic studies carried out in the last eight years followed by ABI SOLiD. The longer read length resulting due to Roche chemistry allows unambiguous mapping of reads to complex targets, giving Roche 454 platform an upper edge over other competitors. The another major player Illumina's (earlier Solexa) offerings, HiSeq 1500/2500, HiSeq 2000/1000, and Genome Analyzer IIX are widely used NGS platforms for metagenomic research. One of the latest additions of Illumina, that is, HiSeq 1500/2500, offers two run modes (rapid run and high throughput run mode). This high throughput run mode is perfect for larger studies with more samples and hence is best suited for metagenomics investigations. It requires only 1 ng of community DNA to get complete metagenomic sequence data using reversible terminator chemistry of Illumina for their HiSeq 2500 which is able to generate 270–300 GB of sequence data with read length of up to 200 bp and very high coverage in a short period of less than 5 days. Illumina's recently launched NGS platform HiSeq X Ten has more than 1.5 Tb data output with more than 3 billion reads (above 150 bp size) per flow cell. After Roche 454 and Illumina's NGS platforms, the polony sequencing based ABI (now Life Technologies) SOLiD platforms with highest accuracy (99.99%) are frequently applied in metagenomic research. These NGS platforms are amenable for deep sequencing which makes it possible to detect very low abundant members of complex populations in metagenomic samples. The actual read length and depth required will depend on the desired sensitivity and complexity of the population. NGS technologies have led the way for shotgun metagenomics to reconstruct whole bacterial and archaeal genomes without presence of a reference genome (or their genome sequence) by using powerful assembly algorithms that join short overlapping DNA fragments generated by the NGS sequencers. As each NGS platform differs substantially in read length, coverage, and accuracy, whether these platforms recover the same diversity from a sample remains a fundamental question. Luo et al. [61] carried out direct comparison of the two most widely used NGS platforms, that is, Roche 454 FLX Titanium and Illumina Genome Analyzer (GA) II, on the same DNA samples obtained from Lake Lanier, Atlanta. They inferred ~90% assembly overlap of total sequences and high correlation (*R* ^2^ > 0.9) for the* in situ* abundance of genes and genotypes between two platforms and sequence assemblies produced by Illumina were of equivalent quality to Roche 454 as evaluated on the basis of base call error, frame shift frequency, and contig length. Ion Torrent (and more recently Ion Proton), Pacific Biosciences (PacBio) SMRT sequencing, and Complete Genomics offering DNA nanoball sequencing are few other emerging sequencing technologies, but none of these emerging sequencing technologies have been thoroughly applied and tested with metagenomic samples. NGS platforms are amenable to multiplexing where hundreds to thousands of samples can be sequenced in parallel by adding 9–12 bp DNA tag to each DNA fragment prior to sequencing. Later, this tag is used to identify the origin of the fragment from pooled samples permitting the simultaneous exploration of thousands of bacterial communities in a highly cost-effective manner [62].

The sequence reads generated in NGS based sequencing are typically shorter (except for Pacific Biosciences) than traditional Sanger sequencing reads and have origin from genome of different organisms, which makes the assembly and analysis of metagenomic NGS sequence data extremely challenging. Apart from the problem of assembly of short DNA sequence reads, terabyte-sized data files are generated with each run of instrument, which greatly increases the computer resource requirements of the sequencing laboratories. In a typical sequencing based metagenomic project, postsequencing steps such as metagenomic sequence assembly, functional annotation, binning of sequences, variant analysis, gene/ORF prediction, community taxonomic profile, and metabolic reconstruction are the most critical steps which decide the outcome of any investigation. The majority of current assembly programs are designed to assemble the sequences coming from single genome and hence not equally effective for a typical metagenomic sequence data set having sequences of different origin. Absence of any reference genome for assembly of genome sequences from unculturable representatives of metagenomic sequence pool makes the task more challenging.

Although several bioinformatics tools for sequence assembly of sequences of metagenomic origin have been developed in past few years, which have simplified the task to some extent, still postsequencing analysis is most challenging. Constant efforts are underway to improve the accuracy of alignment of NGS data in several laboratories all across the globe. Development of sequence assemblers like MetaVelvet [63] and Meta-IDBA [64] which are specifically designed for* de novo* assembly of metagenomic sequence reads and metagenomic analysis and data storage pipelines such as MG-RAST [65], MetAMOS [66], MEGAN, IMG/M [67], CAMERA [68], and GALAXY web server [69] has enabled the researchers with limited expertise in bioinformatics to undertake elaborative projects in metagenomics. A brief account of these bioinformatics tools commonly used for postsequencing analysis of metagenomic data is described in Table 3, in order to provide instant information for researchers having limited expertise in bioinformatics.

Longer read length results in better assembled contigs, which further results in quality scaffolds. Sequencing errors remain major issue and extent of sequencing error is different for different sequencing platforms as mismatches are reported more frequently on Illumina platform, and homopolymer issues resulting in insertion/deletions are often reported with Roche 454 platform. Intrinsic sequencing coverage bias of different platforms can complicate subsequent analysis. There exists no gold standard for metagenomic data analysis and inadvertent errors have to be taken care of at each core step of metagenomic investigation.

Currently, there exist simulation systems (GemSIM [70], MetaSim [71], and Grinder [72]) for NGS sequencing data and they can be applied for metagenomic simulation. MetaSim and Grinder use fixed probabilities of sequencing errors (insertions, deletions, and substitutions) for the same base in different reads, but sequencing coverage biases are not considered by any of these simulators. Jia et al., 2013 [73], have developed Next Generation Sequencing Simulator for Metagenomics (NeSSM) which not only deals with sequencing errors but also deals with sequencing coverage biases effectively. The development of new algorithms for extracting useful information out of metagenomic sequence data is so rapid that new updates and developments are reported every couple of weeks and any comprehensive review of this aspect may appear incomplete due to the continuous upgrade and addition of new algorithms.

## 5. Conclusion and Future Perspectives

Information from metagenomic libraries has the ability to enrich the knowledge and applications of many aspects of the industry, therapeutics, and environmental sustainability. The last two decades witnessed tremendous progress in function driven screening of metagenomic libraries constructed using community DNA from various, moderate to harsh environments resulting in the discovery of many novel enzymes, bioactive compounds, and antibiotics through heterologous gene expression. Availability of methods to extract DNA from almost any kind of environmental samples, rapidly dropping cost of sequencing, continuously evolving NGS platforms, and readily available computing and analytical power of automated metagenomic servers have brought the science of metagenomics to extremely exciting phase. The perfect stage has been set for executing and implementing the accumulated insights about untapped microbial communities to exploit their concealed potential. Metagenomic data sets are increasingly becoming more complex and comprehensive and* in silico* gene prediction on metagenomic sequence data sets is rocketing. After 2005, enormous information about novel genes/ORFs/operons from diverse environments has accumulated. Now, there is strong need to focus more on validating these novel genes/ORFs of metagenomic origin by putting them in action in real wet lab conditions to search for more novel enzymes and bioactivities for bioprospecting metagenomes; else, we may end up putting all efforts for novel genes/ORFs/operons in dry lab conditions only. Systems biology approach combined with Next Generation Sequencing technologies and bioinformatics is inevitable for achieving these objectives.

## Figures and Tables

**Table 1:** Biological functions derived from the metagenomes from diverse habitats.

Type of activity exhibited by the metagenomic clone	Library type	Number of clones screened/size of DNA used for library construction	Sampling site	Screening method	Reference
Lipase	Plasmid and fosmid	29.3 Gb of cloned soil DNA	German forest soil (horizon A)	Phenotypic detection (tributyrin hydrolysis)	[50]
	Fosmid	200,000 clones	Qiongdongnan basin, South China Sea (water depth 778.5 m)	Phenotypic screening (tributyrin hydrolysis)	[51]
	Fosmid	15,000 clones	Peat-swamp forest soil from Narathiwat Province, Thailand	Phenotypic detection (tributyrin hydrolysis)	[52]

Esterase	Plasmid	20,000 clones	High Andean forest soil	Phenotypic detection (tributyrin hydrolysis)	[53]
	Fosmid	20000	Deep-sea sediment	Phenotypic detection (tributyrin hydrolysis)	[54]
	Fosmid	142,900	Red pepper plant rhizosphere and strawberry plant rhizosphere	Phenotypic detection (tributyrin hydrolysis)	[55]

Protease	Fosmid	17000	Surface sand from the Gobi and Death Valley deserts	Phenotypic detection (skimmed milk)	[74]
	Plasmid	70,000	Goat skin surface	Phenotypic detection (skimmed milk)	[75]

Laccase	Plasmid	8000	Mangrove soil	Phenotypic detection (hydrolysis of guaiacol)	[76]
	Phagemid	Not mentioned	Bovine rumen microflora	Phenotypic detection (oxidation of syringaldazine)	[77]

Agarase	Cosmids	1,532	Soil from uncultivated field (Germany)	Phenotypical detection (hydrolysis of low melting point agarose)	[78]

Amidase	Plasmids	193,000	Soil and enrichment cultures from marine sediment, goose pond, lakeshore, and an agricultural field (Netherlands)	Heterologous complementation	[79]

Alcohol oxidoreductase	Plasmids	900,000 and 400,000	Soil and enrichment cultures from a sugar beet field (Germany), river sediment (Germany), sediments from Solar Lake (Egypt), and sediment from the Gulf of Eilat (Israel)	Phenotypic detection (NAD(P)H-dependent reduction of carbonyls or by measuring the NAD(P)-dependent oxidation of alcohols)	[80]

Antibiotics and bioactive compounds with anti-infective properties	Fosmid	80,500 clones from Yuseong and 33,200 clones from Jindong Valley forest soil	Forest soil from Jindong Valley	Phenotypic detection	[81]
	Cosmids	Not mentioned	Bromeliad tank water (Costa Rica)	Phenotypical detection	[82]
	BAC	24,546	Soil	Phenotypic detection	[83]

DNA polymerase 1	Plasmid	21,198 Sanger sequence reads were analyzed	Octopus hot spring (93°C) in Yellowstone National Park	Activity-based screening (primer extension assay)	[84]

Na^+^/H^+^ antiporters	Plasmid	8,000	Chaerhan Salt Lake, China	Heterologous complementation	[85]

Cellulases and xylanases	Fosmid library	Not mentioned	Hindgut of wood-feeding termite	AZCL-HE cellulose and AZCL-Xylan based assay	[86]

Phytases	Fosmid library	14,440	Soil	Functional screening (by supplying only the phytate as the sole P source in the growth medium and selecting only clones with strong growth rate)	[87]

**Table 2:** Comparison of the unique features of NGS platforms widely applied in metagenomic research.

Sequencer	Roche/454 GS FLX Titanium	HiSeq 2000	SOLiDv4
NGS chemistry	Pyrosequencing	Sequencing by synthesis	Sequencing by ligation and exact call chemistry

Library/template preparation	Emulsion PCR (emPCR)	Solid phase amplification	Emulsion PCR for fragment/mate-pair end sequencing

Average read length	250–310 bp (highest among the NGS platforms) Now approaching 400–500 (titanium) pyroreads	Initially it was 36, now approaching 150	35

Run time (days)	24 hours (fastest of all)	4 days (fragment run) 9 days (mate-pair run)	7 days (fragment run) 14 days (mate-pair run)

Output data/run	0.7 Gb	600 Gb (over 1 Tb with Illumina's HiSeq X Ten)	120 Gb

Advantage	Longer reads Least time for one run Amenable to multiplexing allowing many samples in single run	High throughput Most widely used platform	Highest accuracy due to ECC (exact call chemistry)

Limitations	High error rate in homopolymer region High cost of reagents Low in throughput Artificial replicate sequences during ePCR [88]	Short read length Low multiplexing capability of samples Single base error with GGC motifs High error rate at tail end reads [89]	Long run time Short read length

**Table 3:** A brief description of bioinformatic tools commonly employed for postsequencing analysis of metagenomic sequence data.

Postsequencing task	Bioinformatic tool	Brief description	URL	Reference
Metagenomic assembly tool	MetaVelvet	Decomposes a de Bruijn graph into individual subgraphs on the basis of coverage (abundance) difference and graph connectivity. Overcomes the limitation of a single-genome assembler to misidentify sequences from highly abundant species as repeats. Results in higher N50 scores than any single-genome assembler.	http://metavelvet.dna.bio.keio.ac.jp/	[63]
	Meta-IDBA	Implies partitioning the de Bruijn graph into isolated components of different species by grouping similar regions of similar subspecies and partitioning the graph into components based on the topological structure of the graph.	http://i.cs.hku.hk/~alse/hkubrg/projects/metaidba/	[64]
	Genovo	Uses Bayesian approach and generative probabilistic model of read generation which works by discovering likely sequence reconstructions under the model. Algorithm used is iterated conditional modes (ICM) algorithm, which maximizes local conditional probabilities sequentially.	http://cs.stanford.edu/group/genovo/	[90]
	Bambus 2	Uses mate-pair information during the assembly process which is not used by Meta-IDBA, MetaVelvet, and Genovo. Algorithms operate on a contig graph generation followed by orientation, positioning, and simplification for proper scaffolding.	http://amos.sf.net.	[91]

Short read alignment and mapping to reference genome	Bowtie	An ultrafast and memory-efficient tool for aligning sequencing reads to long reference sequences which employs Burrows-Wheeler index based on the full-text minute-space (FM) index having low memory footprint (1.3 GB only) also supports gapped, local, and paired-end alignment modes.	http://bowtie-bio.sourceforge.net/index.shtml	[92]
	BWA	Employed for mapping low-divergent sequences against a large reference genome. Has three-algorithm mode for different read length. For Illumina sequence reads up to 100 bp size algorithm BWA-backtrack is used, while algorithms, BWA-SW and BWA-MEM, meant for longer sequences ranged from 70 bp to 1 Mbp.	http://bio-bwa.sourceforge.net/	[93]
	SOAP 3	Fast, accurate, and sensitive GPU-based short read aligner which delivers high speed and sensitivity simultaneously. Found to take less than 30 seconds to align one million read pairs onto the human reference genome, much faster than BWA and Bowtie.	http://www.cs.hku.hk/2bwt-tools/soap3-dp/	[94]
	mrsFAST	A cache oblivious mapper that is designed to map short reads to reference genome. mrsFAST maps short reads with respect to user defined error threshold.	http://sfu-compbio.github.io/mrsfast/	[95]

Microbial diversity analysis	MLST	Exploits unambiguous nature and electronic portability of nucleotide sequence data for the characterization of microorganisms.	http://www.mlst.net/	[96]
	Axiome	Streamlines and manages analysis of small subunit (SSU) rRNA marker data in QIIME and mothur. Has a companion graphical user interface (GUI) and is designed to be easily extended to facilitate customized research workflows.	http://neufeld.github.com/axiometic	[97]
	PHACCS	Uses the contig spectrum from shotgun DNA based on modified Lander-Waterman algorithm sequence assemblies to predict structure of viral communities and make predictions about diversity.	http://phaccs.sourceforge.net/	[98]

Functional annotation	RAMMCAP	An ultrafast method that can cluster and annotate one million metagenomic reads in only hundreds of CPU hours.	http://weizhong-lab.ucsd.edu/rammcap/cgi-bin/rammcap.cgi	[99]

Gene annotation/gene calling	FragGeneScan	Combines sequencing error models and codon usages in a hidden Markov model to improve the prediction of protein-coding region in short reads.	http://omics.informatics.indiana.edu/FragGeneScan/	[100]
	MetaGeneMark	An ab initio gene prediction tool with updated heuristic models designed for metagenomic sequences.	http://exon.gatech.edu/meta_gmhmmp.cgi	[101]
	MetaGeneAnnotator	Precisely predicts all kinds of prokaryotic genes from a single or a set of anonymous genomic sequences having a variety of lengths. Integrates statistical models of prophage genes in addition to those of bacterial and archaeal genes and also uses a self-training model from input sequences for predictions.	http://metagene.cb.k.u-tokyo.ac.jp/	[102]

Binning	TETRA	Based on statistical analysis of tetranucleotide usage patterns in genomic fragments which automate the task of comparative tetranucleotide frequency analysis and outperform (G+C) content based analysis.	http://www.megx.net/tetra/index.html	[103]
	MetaCluster 5.0	A two-round binning method that separates reads of high-abundance species from those of low-abundance species in two different rounds and aims at identifying both low-abundance and high-abundance species in the presence of a large amount of noise due to many extremely low-abundance species. Uses a filtering strategy to remove noise from the extremely low-abundance species.	http://i.cs.hku.hk/~alse/MetaCluster/	[104]
	Phymm	Uses interpolated Markov models (IMMs) to characterize variable-length oligonucleotides typical of a phylogenetic grouping.	http://www.cbcb.umd.edu/software/phymm/	[105]

Automated platforms/servers for comparative and functional analysis of metagenomic sequence data	MG-RAST	MG-RAST (the Metagenomics RAST) server is an automated analysis platform which provides upload, quality control, automated annotation, and analysis for prokaryotic metagenomic shotgun samples.	http://metagenomics.anl.gov	[65]
	MetAMOS	An open source and modular metagenomic assembly and analysis pipeline leveraging over 20 existing tools with some new tools integrated as well. Entire pipeline is built around the unique features provided by the metagenomic scaffolder Bambus 2.	https://github.com/treangen/MetAMOS	[66]
	MEGAN 4	Released in 2011 for taxonomic analysis, comparative analysis, and functional analysis methods based on the SEED and KEGG (Kyoto Encyclopedia for Genes and Genomes)	http://www-ab.informatik.uni-tuebingen.de/software/megan	[106]
	IMG/M	A data management and analysis system for microbial community genomes (metagenomes) hosted at the Department of Energy's (DOE) Joint Genome Institute (JGI). IMG/M consists of metagenome data integrated with isolate microbial genomes from the Integrated Microbial Genomes (IMG) system.	http://img.jgi.doe.gov/cgi-bin/m/main.cgi	[67]
	CAMERA	Provides access to raw environmental sequence data, with associated metadata, precomputed annotation, and analyses. Integrates tools for gene prediction and annotation, clustering, assembly sequence quality control, functional and comparative genomics applications, and many other downstream analysis tools.	http://camera.calit2.net	[68]
	GALAXY	A publicly available web service, with software system that provides support for analysis of genomic, comparative genomic, and functional genomic data through a framework that gives experimentalists simple interfaces to powerful tools while automatically managing the computational details.	http://galaxyproject.org	[69]
